# An automated workflow for enhancing microbial bioprocess optimization on a novel microbioreactor platform

**DOI:** 10.1186/1475-2859-11-144

**Published:** 2012-10-31

**Authors:** Peter Rohe, Deepak Venkanna, Britta Kleine, Roland Freudl, Marco Oldiges

**Affiliations:** 1 Institute of Bio- and Geosciences, IBG-1: Biotechnology, Systems Biotechnology, Forschungszentrum Jülich GmbH, 52425, Jülich, Germany; 2 Institute of Bio- and Geosciences, IBG-1: Biotechnology, Systemic Microbiology, Forschungszentrum Jülich GmbH, 52425, Jülich, Germany

**Keywords:** Microbioreactor, Biolector, Bioprocess development, Strain screening, Heterologous protein expression, Automation, Liquid-handling

## Abstract

**Background:**

High-throughput methods are widely-used for strain screening effectively resulting in binary information regarding high or low productivity. Nevertheless achieving quantitative and scalable parameters for fast bioprocess development is much more challenging, especially for heterologous protein production. Here, the nature of the foreign protein makes it impossible to predict the, e.g. best expression construct, secretion signal peptide, inductor concentration, induction time, temperature and substrate feed rate in fed-batch operation to name only a few. Therefore, a high number of systematic experiments are necessary to elucidate the best conditions for heterologous expression of each new protein of interest.

**Results:**

To increase the throughput in bioprocess development, we used a microtiter plate based cultivation system (Biolector) which was fully integrated into a liquid-handling platform enclosed in laminar airflow housing. This automated cultivation platform was used for optimization of the secretory production of a cutinase from *Fusarium solani pisi* with *Corynebacterium glutamicum*. The online monitoring of biomass, dissolved oxygen and pH in each of the microtiter plate wells enables to trigger sampling or dosing events with the pipetting robot used for a reliable selection of best performing cutinase producers. In addition to this, further automated methods like media optimization and induction profiling were developed and validated. All biological and bioprocess parameters were exclusively optimized at microtiter plate scale and showed perfect scalable results to 1 L and 20 L stirred tank bioreactor scale.

**Conclusions:**

The optimization of heterologous protein expression in microbial systems currently requires extensive testing of biological and bioprocess engineering parameters. This can be efficiently boosted by using a microtiter plate cultivation setup embedded into a liquid-handling system, providing more throughput by parallelization and automation. Due to improved statistics by replicate cultivations, automated downstream analysis, and scalable process information, this setup has superior performance compared to standard microtiter plate cultivation.

## Introduction

Determination of quantitative process parameters for optimizing production strains and cultivation conditions are key elements during bioprocess development for almost every new microbial product. Especially the optimization of heterologous protein production plays a major role, e.g. in biocatalytic and biopharmaceutical manufacturing processes. The protein expression itself is influenced by many biological and bioprocess parameters, which are normally defined in early development phases and kept constant during the scale-up to production scale [1]. Influencing factors are the choice of the expression host, the promoter as well as its induction, temperature switches, batch or fed-batch mode and different media compositions, to mention only some of them [2,3]. To optimize a fermentative production process for a new protein, these parameters must be thoroughly chosen, tailored to the target protein and optimized de-novo due to the differences in nature and characteristics of each protein [4-6].

The challenge is that the number of possible parameter combinations exponentially grows with every additional parameter. To give a simple example, 2 parameters with 4 possible values lead to 16 experiments, whereas for 5 parameters in total 1024 experiments are necessary to identify the optimal parameter setting of all combinations. Hence, on the one hand there is a strong demand for significantly increased cultivation throughput in early process development. On the other hand process monitoring and process control should be as high as possible to allow substantial and reliable parameter optimization to ensure scalability of results.

A process optimization in laboratory scale bioreactors providing detailed informative process data is usually accompanied by a low cultivation throughput. In a given timeframe only a few selected process modifications can be tested and the parameter selection process will be often based on empirical knowledge or by trying to adapt a standard process regime for each new heterologous protein. This clearly illustrates that early bioprocess optimization in laboratory scale bioreactors can only provide a limited contribution.

An increase of cultivation throughput by at least two orders of magnitude is achieved quite easily by cultivation in shaking flasks or microtiter plates (MTP) [7,8]. This is usually accompanied by reduced process information over time and loss of well controlled process conditions present in laboratory scale bioreactors. Several approaches to circumvent those disadvantages were investigated in the last years to hit a middle ground between laboratory scale bioreactors and MTP cultivation [7,9-12] with so called microbioreactors (MBR). MBR concepts can be differentiated in two approaches: The top-down approach is the miniaturization and parallelization of stirred tank reactors down to 10-100 mL gaining process information easily scaled to laboratory scale bioreactors [13-15]. Beside this, the bottom up approach uses standard formats like MTP or shaking flasks extended with enhanced oxygen transfer [16], as well as monitoring of main process parameters [17-19]. The miniaturized stirred reactors can be handled automatically e.g. with an integration of the vessels in liquid-handling platforms [15,20,21]. With such a setup, repeated sampling during cultivations for offline biomass and product quantification for example in an adjacent spectrophotometer as described by Knorr et al. [22] can be carried out. In contrast to this, a sampling event from MTP based cultivations with volumes below 2 mL is usually a total harvest of the whole reaction volume [23]. In novel MTP based cultivation systems, the online monitoring allows real time quantification of biomass and fluorescence signals [24], making sampling obsolete for monitoring the expression of fluorescent proteins [25]. Furthermore the achieved online signals can be used to trigger automated pipetting events like addition of initial cell inocula or IPTG solution with automated robotic workstations [21].

In this contribution, we setup such a smart interactive combination of MTP cultivation system (Biolector) and liquid-handling robotics as a versatile bioprocess optimization platform with high cultivation throughput called Jülich Bioprocess Optimization System (JuBOS). The setup was customized with further hardware components, to enable basic microbial workflows including media preparation, inoculation, sampling and dosing events during cultivation. Further downstream processes like cell separation and photometric protein analysis are possible focusing on the optimization of recombinant protein production.

As a relevant application example, the production of cutinase, a lipase from *Fusarium solani pisi* was investigated as model enzyme expressed in *C. glutamicum*, a novel expression host. This Gram-positive, facultative anaerobic bacterium has been successfully developed as production organism for small metabolic intermediates like glutamate and lysine at industrial scale [26,27]. Furthermore *C. glutamicum* possesses both the SEC-secretion and the Twin-Arginin-Translocation (TAT) protein secretion pathways, which are known to secrete unfolded and folded proteins, respectively. In addition, no detectable extracellular proteolytic activity is known for this organism [28], making it suitable for the secretory expression of protease-sensitive proteins. Thus, current research activities deal with the potential of *C. glutamicum* as producer strain for heterologous proteins [29-32]. The decision about the protein secretion pathway used as well as the secretion efficiency depends strongly on the nature of the signal peptide (SP) fused to the target protein. Unfortunately, the efficiency of the SP is not known a-priori, since it is also determined by the combination of SP and the target protein itself [33,34]. Computer based tools for prediction of efficient signal peptide sequences provide usually only limited information and were proven to be incorrect [34]. Thus, experimental screening procedures with each protein of interest are essential to rank the SP’s regarding their performance for the secretory production. With every new combination of SP and target protein bioprocess parameters like media components and induction strategy must be optimized de-novo. To meet the requirements of such process optimizations, we describe in this study the validation and application of a MBR system integrated into a robotic platform. In contrast to former publications [21] one challenge is the demand for offline protein quantification of the non-fluorescent cutinase which is met by additional hardware on the automated platform like MTP centrifuge and MTP reader on the JuBOS.

## Results and discussion

### Sterile environment of the cultivation platform

**Figure 1:**
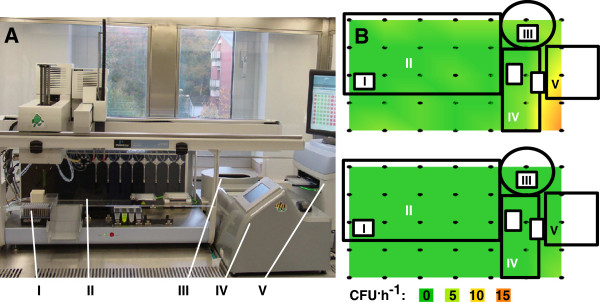
**A: Front view of JuBOS integrated into a cleanbench setup.B**: Top view on robotic platform. 1: Robotic System (JANUS Integrator, PerkinElmer); 2: MTP based cultivation system (Biolector, m2p-labs); 3: Plate Reader (Enspire, PerkinElmer); 4: MTP centrifuge (IXION, Sias); 5: Cooling Adapter (Chillstation, Mécour). Colors indicate colony forming units per hour (CFU^.^h^-1^), top: operating mode, bottom: night mode.

The handling of sterilized cultivation media and solutions during upstream processing steps like media preparation and preculture handling has to be carried out in a sterile environment. To avoid airborne contaminations, the JuBOS is embedded in a laminar downflow housing, in which the air flow can be set to 0.45 m^.^s^-1^ (“*operating mode*”) or to 0.2 m^.^s^-1^ (“*night mode*”). The operational laminar flow conditions of this clean bench can be negatively affected by the installed hardware of the cultivation platform. To check for sterile conditions, 35 LB agar plates were placed for 60 minutes in both modes on the whole deck (dark points in Figure 1, B) and incubated afterwards at 30°C for 18 h. As reference three LB plates were positioned outside the clean bench in the lab environment in three different heights (floor, bench, cupboard) leading to a contamination of 36 ± 6 CFU^.^h^-1^. In contrast, the regular operating mode as well as the less effective night mode showed only very low contamination risk (0-2 CFU^.^h^-1^) for large working areas on the deck (Figure 1B). For the night mode a higher contamination of 5-10 CFU^.^h^-1^ was observed for front right corner, where the MTP spectrophotometer (2) and Biolector (3) are placed, most probably interfering the laminar flow conditions (Figure 1B, bottom). Using the regular operating mode this contamination risk in that corner was reduced to 2 CFU^.^h^-1^ (Figure 1B, top). The results indicate that sufficiently sterile conditions are maintained for the critical working area of the JuBOS under both operational conditions allowing automated antibiotic free media preparation.

### Biological cross contamination between pipetting events

During sampling procedures and downstream processing operations, the steel needles used for pipetting are in direct contact with cell suspension. To avoid biological cross contaminations, the teflon coated steel needles need to be cleaned and free of CFU for contamination critical steps like automated media preparation or dosing events into the cultivation wells.

**Figure 2:**
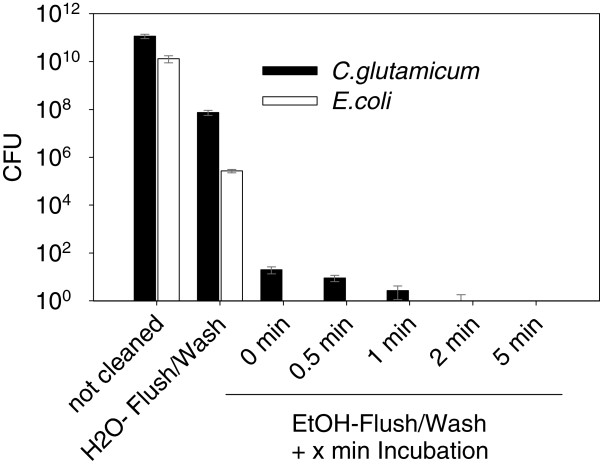
**Biological cross contamination: Colony forming units (CFU) according to needle washing und sterilizing procedures after pipetting of 1000 μL of cell suspension with OD**_**600**_**= 5.**

The robotic setup uses deionized H_2_O as system liquid for the piston stroke as well as for cleaning in place (CIP) protocols. The manufacturer standard protocols include a *flush* step, were a peristaltic pump cleans the inner surface of the needle by pumping 3000 μL H_2_O through each needle into the waste. In the following *wash* step also the outer surface is cleaned by pumping 3000 μL H_2_O into flooded wells of a *washing station*, in which the steel needles are dipped in approx. 5 mm under the liquid surface. Initial experiments with those default CIP protocols to clean the steel needles, contaminated with *C. glutamicum* and *E. coli* (as Gram-negative reference) showed insufficient reduction from approx. 10^10^ to 10^6^ CFU (Figure 2). To enable “sterilization in place” (SIP) of the steel needles during an automated protocol a three way valve connects a tank of 70% v/v EtOH with the system tubing. After an ethanolic flush/wash-step without an ethanol incubation of the tubing system no CFU were found for *E. coli* and only for Gram-positive *C. glutamicum* around 10 CFU were found. This contamination can be completely eliminated after incubation for 5 min proving proper pipetting conditions for *C. glutamicum* without risk of cross contamination. Consequently, for contamination critical steps, like antibiotic free media preparation, the pipetting needles must be incubated for 5 minutes in 70% ethanol.

For other pipetting procedures which are done in later process phases, like induction with IPTG or sampling events, the strict necessity to avoid any carry-over of cells from one culture to another culture is not prevalently critical. The transfer of at least 10 *C. glutamicum* cells still present after quick ethanolic flushing will have no effect in the presence of a biomass concentration of at least 10 g^.^L^-1^, which corresponds to about 10^11^ CFU^.^L^-1^. This clearly shows the negligible impact of the cross-transferred cells compared to the 10^10^ times higher cell concentration in the cultivation. Thus, it seems to be a reasonable trade-off between sterility issues and speed to choose the faster variant of cleaning the steel needles if indicated. For automated media preparation this is no choice, since even one single contaminant cell will spoil the experiment.

### Automated media preparation with low statistical error

The ability to perform the upstream processing in an automated fashion under sterile conditions allows preparing the cultivation media for the small scale cultivation directly on the robotic deck prior to cultivation. In most applications of parallel small scale cultivations the MTP for cultivation is filled manually instead of doing this automatically [35,36]. Even for higher throughput this is typically not a limiting step, since it can be achieved quickly using multi-channel pipettes or semi-automated lab dispensers if the same medium is used for all cultivations.

The media preparation becomes a time-critical step, if a diverse set of media compositions has to be prepared, e.g. to investigate the influence of different media components on the production performance of the microbial system. In such cases, the automated media preparation is highly advantageous, since the robotic system 1) shows superior speed in pipetting different media compositions from stock solutions and 2) provides higher reliability of pipetted medium composition accompanied by an electronic documentation of the performed pipetting events.

The automated media preparation with the JuBOS was first validated against the manually prepared medium. Therefore, the minimal medium CG XII for *C. glutamicum* was prepared by the JANUS workstation from four stock solutions containing single components ((NH_4_)_2_SO_4_, Urea, ZnSO_4_, CuSO_4_) in 20 fold concentration and one further stock solution containing all the other components of CG XII medium in twofold concentration. The required volumes of stock solutions were pipetted into the wells of the cultivation plate (i.e. flowerplate) in fourfold replicates and were automatically filled up to the start volume of 950 μL with sterile deionized water. The manually pipetted medium was prepared from the same stock solutions with a final volume of 42 mL and 950 μL was dispensed in four replicates into the Flowerplate. All cultivation wells were inoculated with 50 μL preculture.

**Figure 3:**
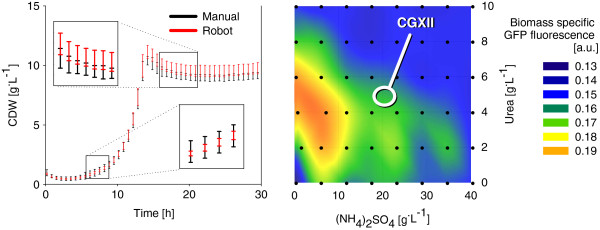
**A: Growth characteristics of *C. glutamicum *cultivated in manually and automatically prepared CG XII media each with four replicates.B**: Variation of the two nitrogen sources (NH_4_)_2_SO_4_ and urea showed significant differences of GFP fluorescence used as signal for protein expression. Black dots in the contour plot represent the 48 investigated mixtures.

The comparison of the growth characteristics between the manual (μ = 0.37 ± 0.01 h^-1^; CDW_Max_ = 11.4 ± 0.6 g^.^l^-1^) and automated (μ = 0.36 ± 0.01 h^-1^; CDW_Max_ = 10.7 ± 0.3 g^.^l^-1^) media preparations showed very good similarity (Figure 3A). The relative statistical error of CDW_Max_ (3-5%) is very close to the range of the pipetting accuracy of the JANUS workstation [37]. The slight systematic deviation of the CDW_Max_ between the two sets of replicates cannot be proven as significant with a two sided *t*-test (p = 0.08). Hence, the automated media preparation could be successfully validated against the standard approach of manual preparation. This tool can now be routinely used to facilitate the application of the cultivation platform. Medium recipes to be prepared are easily integrated by simple upload of an excel compatible worklist into the WinPREP software of the robotic workstation enabling robust and convenient media preparation prior to small scale cultivation.

To illustrate the benefit of the option for automated media preparation, we applied this method to investigate the effect of two nitrogen sources, namely (NH_4_)_2_SO_4_ and urea on the protein expression of GFP in *C. glutamicum*. A full factorial design approach was used with variable urea and ammonium sulfate concentration resulting in 48 different media prepared in one MTP by the robotic system. The manual preparation from 4 stock solutions and additional water like described above would take approx. 1.5 hour. This workload is drastically reduced below 10 min by using the automated media preparation proving a fast media preparation method.

The *C. glutamicum* cells contain plasmid pEKEx2-PhoD-GFP that harbors the gene for a GFP protein that is fused to the signal peptide of the *B. subtilis* TAT-substrate PhoD. Expression of the PhoD-GFP fusion in *C. glutamicum* leads to TAT-dependent secretion of the folded GFP protein into the culture supernatant [29]. As comparative signal for protein production, the maximal online fluorescent signal was used and normalized with the maximum biomass signal obtained in each cultivation to achieve a biomass-specific GFP formation (Figure 3B). The data indicate that the concentrations of the nitrogen sources in standard CG XII medium (see marker) do not provide optimal conditions for GFP production. The biomass specific fluorescence is 50% higher at reduced ammonium sulfate concentrations (between 0 to 5 g^.^L^-1^) and an urea concentration at 10 g^.^L^-1^, represented as red area in Figure 3B.

With GFP as an example for protein expression in *C. glutamicum*, the CG XII medium seems to be not ideally suited. In current applications *C. glutamicum* is mainly used for production of small metabolites from primary metabolism, e.g. lysine [38], glutamate [39], valine [40] or succinate [41] and the published cultivation media have been optimized in the past for fast growth and amino acid production [42-44]. But during the last 5 years *C. glutamicum* gains in importance as a host for secretory protein production. The published cultivation medium used for those protein production processes [30,32] is also minimal media derived from CG XII [43] and differ mainly in the added trace metal and CaCO_3_ concentrations. The bulk nitrogen source of both, the CG XII media and the protein secretion media is ammonium sulfate, which shows a negative effect on the fluorescence quantity in this study. Urea appears to be a proper alternative nitrogen source for the production of secretory GFP.

So far, a description of a systematic media optimization for secretory protein production with *C. glutamicum* has not been investigated. Our results indicate clearly a significant influence of the media composition on the overall production. The optimization of the medium specifically suited for a certain microorganism or target application seems to provide a clear benefit. By the use of the JuBOS such effects can be identified and the automation can be efficiently used for routine media optimization during bioprocess development.

### Precise strain characterization by triggered sampling events

The challenge of process related screening experiments in MBR is to increase the experimental throughput while maintaining process relevant conditions and to achieve scalable information about the microbial production characteristics. To keep the throughput in MTP based approaches as high as possible, it is necessary to analyze the characteristic strain parameters in a single cultivation well per clone. A common and easy approach is end-point determination in an “over night cultivation” with a constant cultivation time, synchronous IPTG induction at the beginning and a synchronous harvest of all cultivations wells in the stationary phase [34,45]. We investigated three cutinase producer strains each with a different signal peptid of SEC-substrates from *B. subtilis* (Bpr, YmwC, AmyE). The lipolytic activity and its statistical error are analyzed with 9 to 12 fold biological replicates, each with different colonies as starting material for precultures.

**Figure 4:**
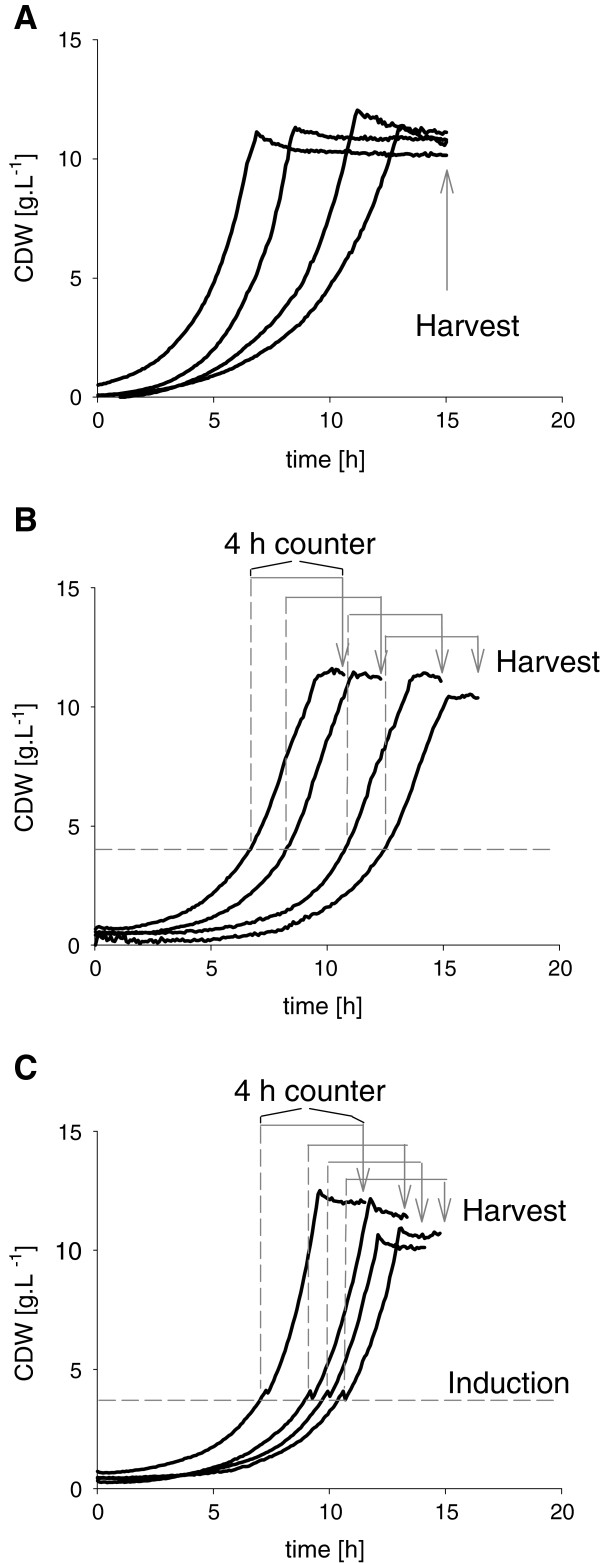
**Three harvest methods exemplarily shown with *C. glutamicum *producing bpr-Cutinase.A**: Synchronous harvest of all cultivation wells in the stationary phase (“over night cultivation”). **B**: Triggered harvest of total reaction volume 4 h after reaching 4 g^.^L^-1^ CDW (BS = 100 a.u.) in early stationary phase. **C**: Triggered induction at 4 g^.^L^-1^ CDW (BS = 100 a.u.) and triggered harvest 4 h later in early stationary phase.

The cultivations in the MTP were harvested completely, when the last cultivation had reached the stationary phase after 15 h. The growth of the three *C. glutamicum* constructs showed no different phenotypes, but a strong variability of the lag phase, as well as differences of μ_Max_ and Y_X/S_ (Figure 4A: for clarity, only 4 growth curves each are displayed, full dataset in Additional file 1 ), probably caused by different vitality of the inoculated preculture suspensions. Those differences lead also to different times of reaching the stationary phase resulting in mean stationary phase duration of 6 h ± 1.4 h (Table 1). The analyzed biomass specific lipolytic activity EA_spec_ of the supernatant has a high relative standard deviation up to 40% (Table 1), which leads to an uncertain strain selection due to the high biological variation. Also other SP screening studies with *B. subtilis* as host organism resulted in an almost similar error of 25% of lipolytic activity, when 8 replicates in MTP cultivation were sampled in the stationary phase [34]. If information solely from stationary phase is used for cell selection, good producer strains might be also undetectable, if the protein is instable and degraded in the stationary phase [46,47]. For proteins very sensitive to instability and degradation associated to stationary phase conditions the standard error is expected to increase. Even fast and effective good producers might be not identified, since the majority of the product is already degraded and lost.

**Table 1:** **Influence of three harvest methods on the lipolytic activity (EA**_**spec**_**) fused with three different signal peptides (bpr, amyE, ywmC)**

	**A: EA**_**spec**_**[kU**^**.**^**g**^**-1**^**] “Over night cultivation”**	**B: EA**_**spec**_**[kU**^**.**^**g**^**-1**^**] Triggered harvest**	**C: EA**_**spec**_**[kU**^**.**^**g**^**-1**^**] Triggered induction + harvest**
bpr	10.11 ± 2.98	11.84 ± 0.96	13.34 ± 1.25
ywm	3.61 ± 1.50	5.31 ± 0.60	4.40 ± 0.54
amyE	1.22 ± 0.64	1.62 ± 0.33	1.49 ± 0.24
Mean relative error	41%	13%	13%
Duration of stationary phase	6.0 h ± 1.7 h	0.9 h ± 0.4 h	1.6 h ± 0.3 h

Error indicate standard deviation of 9 to 12 fold biological replicates (independent clones). Depending on the sampling method, the mean duration of stationary phase is different. Backscatter data of all processes are provided as supplemental data.

Nevertheless, the advantages of MTP cultivations can be used to compare different strains by synchronizing the biomass growth with enzymatic glucose release media from polysaccharides [48-50]. But up to this time, there is no release medium optimized for *C. glutamicum* commercially available. A second opportunity is to equalize the growth behavior by an adjustment of the initial cell number based on OD or backscatter values, which can be carried out by automated robotic systems [51]. With this adjustment the authors achieved a synchronous growth pattern during the batch phase. When such a synchronized MTP was sampled, all cultivations were in the same growth phase leading to a fair comparison of different cultivations. This adjustment is reasonable, when the vitality of the starting cell material and the growth rate in the main culture are similar.

In our study, the precultures were inoculated with cell material from single colonies, then cultivated to stationary phase and transferred to the main culture. Thus, both prerequisites for an adjustment of the initial cell material were not given and a strategy to circumvent the additional burden of culture synchronization would be beneficial.

To achieve reliable data from heterologously growing *C. glutamicum* cultivations, a triggered sampling method was developed on the JuBOS. Depending on the online biomass signal measured by Biolector, each cultivation well was harvested independently 4 h after reaching 4 g^.^L^-1^ CDW in the early stationary phase (Figure 4, B). Cell suspensions were then stored at 4°C in deep well plates and centrifuged at 4000 g for 10 min. With this strategy, the mean duration of stationary phase was lowered to 0.9 h ± 0.4 h. The mean values of EA_spec_ achieved with biomass triggered sampling events are in the same range of the mean EA_spec_ analyzed with synchronous sampling (Table 1). However, the trigger leads to a drastically decreased relative error of 13% compared to 41% which dramatically facilitates the selection of best strains. But this method can be still an unfair comparison, due to the fact, that all cultivations were induced from beginning leading to different induction time for each single cultivation.

To investigate this effect of the induction time, a further opportunity for synchronization of expression pattern was tested. A biomass triggered addition of an inductor substance (e.g. IPTG) leads to a production of recombinant proteins starting always at the same biomass concentration. This method was developed in previous studies and validated with online detectable fluorescent proteins [21]. In our study we combined harvest with this biomass triggered IPTG addition at 4 g^.^L^-1^ CDW in a way that IPTG was added at 4 g^.^L^-1^ CDW and harvesting was done 4 h later (Figure 4C). This was individually done for each cultivation based on the online biomass data fully exploiting the potential of the interactive integration of the cultivation with the automated liquid-handling operation. As it can be seen in Table 1 the lipolytic activities of the three constructs show the same ranking like in the other two methods. Interestingly, with this high sophisticated method, a relative error of 13% can be achieved, which is the same error of the method without triggered induction but with induction from beginning. It can be concluded, that synchronization of the growth related harvesting time is more important than synchronization of induction. Reliable data can be achieved by using the triggered sampling strategy with the simple synchronous induction at the beginning of the cultivation. In our case, this method was developed for the screening of signal peptides, which have a significant impact for the development of secretory protein production [34]. Because of the flexible trigger setpoints, this sampling method is also suitable for any other strain characterization or the investigation of different culture conditions.

### Profiling of optimal induction conditions

In recombinant protein production processes the induction in later process phases is usually beneficial to avoid inhibiting effects like toxicity or metabolic burden. The main parameters that can be influenced in the cultivation process are the concentration of the inductor and the time point of addition. Simultaneous optimization of both parameters in a microtiter cultivation setup proved to be a fruitful approach during the production of fluorescent proteins [21]. The optimal induction strategy not only depends on the coding plasmid and its promoter, but also on the target protein itself [5]. For this reason, a method for induction profiling, was setup analyzing activity of the cutinase enzyme, which is the heterologous target protein to be secreted in *C. glutamicum*.

**Figure 5:**
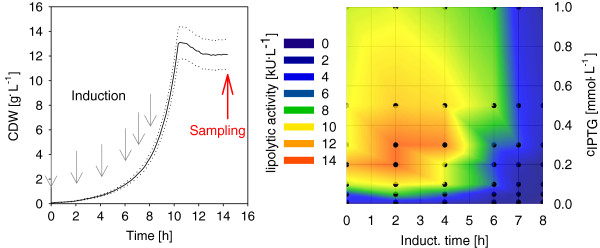
**Induction profiling.A**: CDW of a MTP culture of *C. glutamicum* expressing NprE- Cutinase in CG XII (20 g^.^L^-1^ Glucose). Arrows indicate induction and sampling of 48 cultivations, dotted lines indicate standard error of 10%. **B**: Lipolytic activity in supernatant of sampled MTP cultures. Induction was carried out by adding 25 μL of different concentrated IPTG stock solutions leading to the indicated IPTG concentrations in the MTP cultivations.

In this experiment, the effect of IPTG concentration and induction time on the cutinase production was investigated with the JuBOS. Secretory NprE-cutinase was chosen to be used as model protein produced with *C. glutamicum*, due to improved secretion performance. At six time points, indicated by black arrows in Figure 5A, different concentrations of IPTG were added automatically by the liquid-handling system. This setup leads to a full factorial design for induction optimization in one single flowerplate consisting of 48 cultivations synchronously harvested after 14 h (red arrow, Figure 5A). Supernatant was analyzed for cutinase activity, resulting in contour plot for secretory cutinase of each cultivation (contour plot in Figure 5B).

The highest cutinase production of 13.5 ± 1.4 kU^.^L^-1^ was observed at low IPTG concentrations (0.2 - 0.3 mmol^.^L^-1^) added in the early exponential growth phase. When using the IPTG concentration of 1 mmol^.^L^-1^, which is commonly used for lac based promoters, the measured cutinase activity is lowered drastically by nearly 30% from 13.5 kU^.^L^-1^ to 9.2 ± 0.9 kU^.^L^-1^. Thus, the data show a clear influence of the induction strategy on production behavior of secretory cutinase. Consequentially using this method as an early stage module followed by validation experiments in 1 L bioreactors is strongly recommendable in future bioprocess development for secretory target proteins.

### Scalable process data achieved in MTPs

Reliable results for early upstream bioprocess development are normally achieved in stirred tank reactors with well-defined environment. To characterize protein production processes in the MTP cultivation system Biolector, the obtained growth and production behavior has to be validated in comparison to those bioreactors with commonly used scales like 1 L and 20 L. Here, in all scales *C. glutamicum* producing recombinant cutinase fused to the best performing signal peptide (NprE) of this study was investigated. In 1 mL and 1 L scale two further SPs (YwmC, YpjP) serve as mid and low performance producers to compare the ranking of the SP secretion efficiency. Protein expression was induced at the beginning of the cultivation with 0.5 mM IPTG. Sequential samples were taken every hour, obtaining OD, CDW and lipolytic activity.

In flowerplate cultivations the same preculture, temperature, inoculum size and induction strategy is used. Since no pH regulation can be achieved in MTPs, the medium is buffered with MOPS to an initial pH = 7. The scale-up criterion was a DO > 20% according to Riesenberg et al. [52]. In bioreactors this was achieved by a closed-loop strategy of stirrer and gas flow rate. In flowerplates this criterion was fulfilled at shaking frequency n = 1200 min^-1^ and filling volume V = 1 mL leading to an OTR_Max_ = 80 mmol^.^L^-1.^h^-1^ (information kindly provided by m2p-labs, Aachen/D).

**Figure 6:**
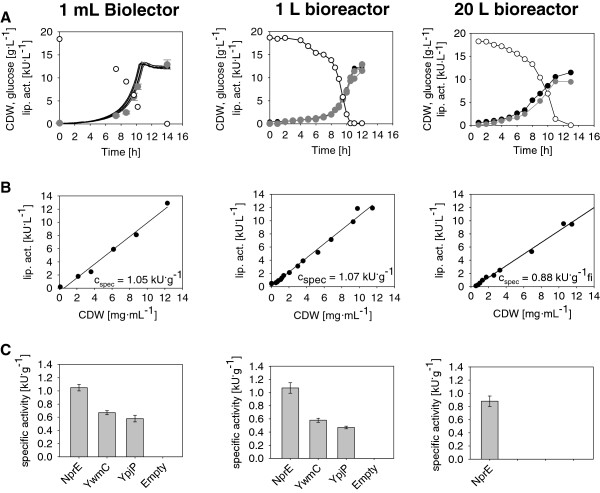
**Scale- up of *C. glutamicum *batch culture.** Left column: Microtiter cultivation (8 replicates, cells harvested for offline analytic). Middle: 1 L lab-scale bioreactor (2 replicates). Right: 20 L technical scale bioreactor (no replica). Secretory NprE- Cutinase is produced with pEKEx2 vector and 0.5 mM IPTG induction at t = 0. Row **A**: CDW (black), Glucose (white dots) and lipolytic activity (grey dots) over time. **B**: lipolytic activity of cutinase over CDW. Slope of regression line represents secretory specific activity EA_spec_ during growth coupled protein expression, **C**: Specific activity EA_spec_ of cutinase expressed with different signal peptides, error bars is the confidence interval of linear regression (see row **B**).

The biomass growth in all three scales (Figure 6A) resulted in the same Y_X/S_ = 0.6 ± 0.05 g_CDW_^ .^g_Glc_^-1^. The maximum specific growth rate of μ_Max_ = 0.41 ± 0.02 h^-1^ was the same in 1 mL MBR as well as in 1 L Bioreactor. Here, the online backscatter measurement in the Biolector was suitable to obtain biomass related parameters (μ_Max_, Y_X/S_). For analyzing protein production parameters like the biomass specific enzyme activity EA_Spec_ [kU^.^g_CDW_^-1^] cell suspension was analyzed offline in enzyme activity assays. For this purpose, parallel 1 mL cultivation wells were harvested iteratively, centrifuged and analyzed with enzyme activity assay. The measured lipolytic activity shows a growth coupled cutinase secretion, just as in 1 L bioreactors, both with an EA_Spec_ ≈ 1 kU^.^g_CDW_^-1^ (Figure 6B). In the 20 L stirred tank reactor both, the EA_spec_ was about 15% lower than in Biolector and the 1 L bioreactor. Because the 20 L cultivation was inoculated on another day with another preculture as the 1 L and 1 mL cultivations, this difference might be attributed by the biological variation in the seed culture. In contrast to this, the 1 mL MTP cultivation process was perfectly comparable to 1 L bioreactor. Furthermore three clones each with different cutinase productivity based on the fused signal peptide were investigated (nprE, ywmC, ypjP). Here the three producer strains show the same ranking of EA_spec_ in 1 L stirred tank reactors and 1 mL MTP cultivations (Figure 6C). Thus, Biolector is well suited for screening experiments resulting in process relevant ranking of different strains with different phenotypes. Together with the triggered sampling events described above, the JuBOS system can analyze strain characteristics in higher through put e.g. during SP screening or IPTG profiling which are directly scalable to 1 L and 20 L stirred tank reactors.

## Conclusion

The progress in MTP based microbioreactor techniques during the last five years has resulted in the easy integration of benchtop instruments with automated liquid-handling stations. The shown validation tests of the Jülich Bioprocess Optimization System (JuBOS) support the usability of such an automated platform for microbial cultivation experiments in high-throughput approach. A complete workflow from sterile media preparation and scalable cultivations to triggered sampling and subsequent at-line analysis of enzyme activity was established for optimization of recombinant cutinase production. Although the cultivation platform was currently used in batch mode only, it is the basis for fed-batch application in the near future employing fed-batch by use of microfluidic technology [53] or slow release systems for substrates [49].

As a key advantage, the resulting statistical error of productivity data of non fluorescent target protein was drastically decreased from 41% to 13%, due to triggered sampling events to synchronize growth pattern of biological replicates. With these methods it is now possible not only to select production strains in a higher resolution but also to optimize reaction conditions like induction strategies and media components based on reliable results that are directly scalable to stirred tank bioreactors.

The throughput is drastically increased due to 48 parallel cultivation wells of the Biolector. For example, the induction profiling carried out on the JuBOS has a process time of approx. 24 h (6 h preculture, 14 h main culture, 2 h DSP) with a manual workload of approx. 2 h. In comparison to this, 48 induction experiments in a 4 parallel bioreactor system (e.g. DASGIP BioBlock) would have a process time of 312 h (12 runs * [6 h setup time & preculture, 14 h main culture, 6 h DSP & cleaning]). This 13 fold increased throughput might seem an unfair comparison, because usually induction or media optimization experiments are carried out in shaking flasks or standard MTP techniques to increase the throughput. Those approaches however often involve cost intensive iteration steps backwards after failing of scale-up from bench to pilot- or production-scale. With our contribution we can close this gap with a flexible and easy to use automated platform for medium throughput cultivation in the early bioprocess development of fermentative protein production processes.

## Methods

### Strains and media

*C. glutamicum* ATCC 13032 was used as host strain. *Gfp*_*UV*_ gene was ligated to *phoD*-SP [29]. Cutinase gene from *Fusarium solani pisi* was ligated to DNA sequences of *nprE*, *ywmC* and *ypjP* SEC- signal peptides [34]. Constructs were cloned into pEKEx2 plasmid using *E. coli* XL1 blue [54]. Cells were transformed via electroporation, selected on brain heart infusion (BHI) agar plates (37 g^.^L^-1^ BHI, 91 g^.^L^-1^ sorbitol, 50 mg^.^L^-1^ kanamycin) and cultivated in BHI boullion (37 g^.^L^-1^ BHI). Cells of exponential growth phase were harvested and incubated in 30% v/v glycerol for 10 min at 20°C and stored in 2 mL aliquots at −80°C. LB agar plates for contamination studies were prepared like described, but without selection marker kanamycin.

Precultures were performed in BHI medium. For all main cultures minimal media CG XII was used [43] with 20 g^.^L^-1^ (NH_4_)_2_SO_4_, 5 g^.^L^-1^ Urea, 1 g^.^L^-1^ KH_2_PO_4_, 1 g^.^L^-1^ K_2_HPO_4_, 13.25 mg^.^L^-1^ CaCl_2_ × 2H_2_O, 0.25 g^.^L^-1^ MgSO_4_ × 7H_2_O, 0.2 mg^.^L^-1^ biotin, 30 mg^.^L^-1^ protocatechuic acid, 10 mg^.^L^-1^ FeSO_4_ × 7H_2_O, 10 mg^.^L^-1^ MnSO_4_ × H_2_O, 1 mg^.^L^-1^ ZnSO_4_ × 7H_2_O, 0.313 mg^.^L^-1^ CuSO_4_ × 5H_2_O, 0.02 mg^.^L^-1^ NiCl_2_ × 6H_2_O. The pH was set to 7.0 with 4 M NaOH. In MTP cultivations 42 g^.^L^-1^ MOPS was used as buffer reagent. Additionally 25 mg^.^L^-1^ kanamycin and up to 0.5 mM IPTG were added. All chemicals were of analytical grade and supplied by Sigma Aldrich.

### Cultivation conditions

All MTP cultivations were carried out in 48 well Flowerplates^TM^ (Art.-No: MTP-48-B, m2p-labs, Aachen/D) incubated in a Biolector device (m2p-labs, Aachen/D). Initial cultivation volume was set to 1 mL per well, including the inoculums of 50 μL. Following cultivation conditions were set in the incubation chamber: shaking diameter 3 mm, frequency 1200 min^-1^, temperature 30°C and relative humidity 80%. MTPs were sealed with gas permeable membranes (Art.-No: F-GP-10, m2p-labs, Aachen/D) to minimize evaporation. For the given shaking diameter (3 mm) and frequency the manufacturer reported a OTR of 0.08 mol^.^L^-1.^h^-1^ which is very close to values achieved in standard stirred tank bioreactors [55,56]. Biomass, dissolved oxygen concentration and pH were measured online in each cultivation well with measurement cycle of 10 minutes. Biomass was detected in the Biolector with backscatter measurement at 600 nm and calibrated like described below. Dissolved oxygen concentration as well as pH values were measured by pre- calibrated optodes at the bottom of each well. Precultures for one strain were carried out in 100 mL two baffled shake flasks with 10 mL media. Multi-strain precultures were carried out with 200 μL media in black 96 well microtiter plates (Greiner Bio-One, Frickenhausen/D) were used with 200 μL media, shaken with 900 min^-1^ and 1.5 mm eccentricity on a Titramax 100 (Biotest, Dreieich/D).

For bioreactor cultivations 2 L DASGIP glass bioreactors (d_I_ = 110 mm, H = 245 mm) equipped with three steel baffles and two six-bladed impellers (d = 45 mm) were used. Dissolved oxygen (DO) was set above 30% with a sequential feedback regulation cascade of stirrer speed (800–1500 rpm) and gas flow (60 L^.^h^-1^ and 120 L^.^h^-1^). Offgas content of CO_2_ and O_2_ was measured with gas analysis (DASGIP, Juelich/D). The pH was set to 7 and controlled with 4 M HCl and 4 M NaOH. Starting volume for all processes was 1 L. Addition of anti foam was controlled by a foam probe. Every hour 5 mL was drawn. Calibration of the peristaltic pumps and recording and controlling of process parameters was done with the process control software DASGIP Manager (DASGIP, Juelich/D).

For scale- up experimentation a 30 L Chemap bioreactor (d_I_ = 260 mm, H = 560 mm) equipped with two six bladed impellers (d = 90 mm) was used. A process control system (iCCC 2000, PCS Systemtechnik GmbH, Munich/ D) enables pH control and DO control with a cascade of stirrer speed (400 min^-1^ to 950 min^-1^) , gas flow rate between 850 L^.^h^- 1^ to 1800 L^.^h^- 1^ and up to 0.8 bar overload pressure. Inoculation was carried out in all scales with 5% v/v vital preculture. 20 mL samples were drawn every hour to quantify biomass, substrate and product concentration.

### Enzyme assay

The detection of the lipolytic activity of cutinase was carried out with the *p*-nitrophenyl-palmitate (pNPP) Assay according to [34,57]. The final concentration of the substrate was 0.8 mM. Optical density (λ = 450 nm) was detected in a Genios Platereader (TECAN, Crailsheim/D) every 25 second for 40 minutes with 3 seconds of shaking before every measurement. Lipolytic activity [kU^.^L^-1^ was determined from values of OD_450_min^-1^. Samples were diluted with deionized water till values of OD^.^min^-1^ > 0.1. Samples with water instead of enzyme solution were used as blanks. The enzymatic activity was calculated using a molar extinction coefficient of 15 cm^2.^μmol^-1^ according to [34].

### Biomass quantification and backscatter calibration

For the determination of cell dry weight (CDW) 1 mL cell suspension was centrifuged at 13000 g for 10 min in predryed and weighed 1.5 mL eppendorf cups. Supernatant was discarded and after pellet resuspended in 1 mL 0.9% w/v NaCl solution and centrifuged again at 13000 g for 10 minutes. After drying at 80°C for 18 h the mass difference was used to calculate CDW. For measurement of OD_600_, samples were diluted with 0.9% NaCl below OD_600_ = 0.8 measured in a Pharmspec UV 1700 photometer (Shimadzu, Duisburg/D).

Backscatter measurement was calibrated in Flowerplates sealed with a gas-permeable membrane (m2p-labs, Aachen/D) at V = 1000 μL, gain = 20, shaking speed 1200 rpm. The backscatter calibration analysis was done with a dilution series of *C. glutamicum* biomass suspension in a range of 0 to 20 g^.^L^-1^ CDW leading to the linear regression equation of CDW = 0.48*BS g^.^L^-1.^au^-1^ - 0.78 g^.^L^-1^ with R^2^ > 0.98. The calibration was confirmed with samples of 1 L batch bioreactors, whose backscatter and CDW were measured like described above.

### Robotic platform for microbioreactor cultivation

Core instrument of the platform is the liquid-handling station JANUS Integrator with a 400 mm overhanging extension (PerkinElmer, Waltham MA/USA) in two arm configuration consisting of a robotic pipetting arm (Varispan^TM^) equipped with 8 steel needles for liquid transfer up to 1.2 mL and a gripper arm for MTP transport on the platform. Liquid-handling procedures are conducted by pumping deionized water as a system liquid through the tube system attached to the steel needles. The system liquid can be switched by an automated 3 way valve (Bürkert, Ingelfingen/D) to 70% v/v ethanol for cleaning procedures to avoid biological cross contamination. The MTP based cultivation system Biolector (m2p-labs, Aachen/D) is installed on the robotic deck in a way that the MTP plate is fully accessible, allowing automated transfer of liquid, i.e. dosage or sampling, as well as replacement of the complete MTP plate. The whole setup is installed in a laminar flow hood (Cleanair, Woerden/NL) with a *high-efficiency-particulate-air-*filter (HEPA-filter) generating sterile environment on the robotic deck. Program automation protocols and methods were written in the command software WinPrep (Version 4.6, PerkinElmer) to manage the required actions of the liquid-handling robotic system. The software Robolector agent V.1.2 (m2p-labs, Aachen/D) organizes the communication between WinPrep software and the Biolector. Cultivation data measured online by the Biolector (biomass, pH, DO) can be used as trigger signals to automatically initiate pipetting events like sampling and dosing for each single cultivation well independently.

The sampled cell suspension is stored in a deep well plate (DWP) placed on a cooling rack (MeCour, Groveland MA/USA) connected with a Cryostat Unichiller (Huber, Offenburg/D) in a temperature range beween - 10°C and + 20°C on the robotic deck. A MTP centrifuge (IXION, Sias/CH) is also fully accessible for the robotic system. Supernatant was separated in DWPs via centrifugation at 4000 g for 15 minutes.

### Sterility test of steel needles

1000 μL of exponential growing cell suspension (OD_600_ = 5) are aspirated and released again. Then different treatments like washing with water, EtOH and incubating with EtOH were investigated along with positive control without any washing. Afterwards 50 μL of the upcoming system liquid was pipetted into 1000 μL VE- Water and CFU was determined on LB agar plates.

## Abbreviations

a.u.: Arbitrary unit; BS: Backscatter; CIP: Cleaning in place; CDW: Cell dry weight; CFU: Colony forming units; DO: Dissolved oxygen; DSP: Downstream processing; DWP: Deepwell plate; EA: Enzyme activity; EtOH: Ethanol; IPTG: Isopropyl β-D-1-thiogalactopyranoside; GFP: Green fluorescent protein; MBR: Microbioreactor; MTP: Microtiter plate; OD: Optical density; SIP: Sterilizing in place; SP: Signal peptide.

## Competing interests

The authors have declared that no competing interest exists.

## Authors’ contribution

PR designed the presented platform, developed and validated the experimental methods and prepared the manuscript. DV performed the scale-up studies and supported with manuscript preparation. MO initiated the project and is the principal investigator, supported with conception and manuscript preparation. BK and RF constructed the secretory producer strains, supported with data interpretation and manuscript preparation. All authors read and approved the final manuscript.

## Supplementary Material

Additional file 1 **Table S1.** Backscatter data (gain 20): Synchronized induction and sampling (Over-night-culture) of C.glutamicum producing cutinase with three different SP: bpr (Well 1-9), YwmC (Well 10-18), AmyE (Well 19-27). **Table S2:** Backscatter data (gain 20): Synchronized induction and triggered sampling of C.glutamicum producing cutinase with three different SP: bpr (Well 1-10), YwmC (Well 11-20), AmyE (Well 21-31). **Table S3:** Backscatter data (gain 20): Triggered Induction and triggered sampling of C.glutamicum producing cutinase with three different SP: bpr (Well 1-12), YwmC (Well 13-24), AmyE (Well 15-36), Well 37-44: C.glutamicum with pEKEX2:Empty Vector. Click here for file
